# Anthocyanin-Enriched Riceberry Rice Extract Inhibits Cell Proliferation and Adipogenesis in 3T3-L1 Preadipocytes by Downregulating Adipogenic Transcription Factors and Their Targeting Genes

**DOI:** 10.3390/nu12082480

**Published:** 2020-08-17

**Authors:** Phutthida Kongthitilerd, Tanyawan Suantawee, Henrique Cheng, Thavaree Thilavech, Marisa Marnpae, Sirichai Adisakwattana

**Affiliations:** 1 Interdisciplinary Program of Biomedical Sciences, Graduate School, Chulalongkorn University, Bangkok 10330, Thailand; phutthida.ko@gmail.com; 2 Phytochemical and Functional Food Research Unit for Clinical Nutrition, Department of Nutrition and Dietetics, Faculty of Allied Health Sciences, Chulalongkorn University, Bangkok 10330, Thailand; tanyawan.s@chula.ac.th (T.S.); mmarisa.hsc@gmail.com (M.M.); 3 Department of Comparative Biomedical Sciences, School of Veterinary Medicine, Louisiana State University, Baton Rouge, LA 70803, USA; hcheng@lsu.edu; 4 Department of Food Chemistry, Faculty of Pharmacy, Mahidol University, Bangkok 10440, Thailand; thavaree.thi@mahidol.ac.th; 5 The Halal Science Center, Chulalongkorn University, Bangkok 10330, Thailand

**Keywords:** Riceberry rice, anthocyanin, cell proliferation, adipogenesis, preadipocytes, obesity, 3T3-L1 cells

## Abstract

Riceberry rice (*Oryza sativa* L.) is a new pigmented variety of rice from Thailand. Despite its high anthocyanin content, its effect on adipogenesis and adipocyte function remains unexplored. We investigated whether Riceberry rice extract (RBE) impacted cell proliferation by examining viability and cell cycle, using preadipocyte 3T3-L1 cells. To test RBE’s effect on adipocyte formation, cells were cultured in adipogenic medium supplemented with extract and adipocyte number and triglyceride levels were quantified. Furthermore, Akt1 phosphorylation along with RT-qPCR and intracellular calcium imaging were performed to obtain an insight into its mechanism of action. The effect of RBE on adipocyte function was investigated using glucose uptake and lipolysis assays. Treatment of cells with RBE decreased preadipocyte number without cytotoxicity despite inducing cell cycle arrest (*p* < 0.05). During adipogenic differentiation, RBE supplementation reduced adipocyte number and triglyceride accumulation by downregulating transcription factors (e.g., PPARγ, C/EBPα, and C/EBPβ) and their target genes (*p* < 0.05). The Akt1 phosphorylation was decreased by RBE but insignificance, however, the extract failed to increase intracellular calcium signals. Finally, the treatment of adipocytes with RBE reduced glucose uptake by downregulating Glut4 mRNA expression and enhanced isoproterenol-induced lipolysis (*p* < 0.05). These findings suggest that RBE could potentially be used in the treatment of obesity by inhibiting adipocyte formation and proliferation.

## 1. Introduction

Obesity is an abnormal condition in which an imbalance between energy intake and energy expenditure occurs, leading to fat accumulation in adipose tissue [1]. It becomes life-threatening and reduces quality of life, as it increases the risk of developing non-communicable diseases (NCDs). In general, obesity is characterized by increased expansion of white adipose tissue, resulting from increased fat-cell size (hypertrophy) and fat-cell number (hyperplasia or adipogenesis) [2]. Adipogenesis rapidly begins with the induction of initiating transcription factor, C/EBPβ mediated by 3-isobutyl-1-methylxanthine (IBMX) and dexamethasone [3]. Then, C/EBPβ activates key adipogenic transcription factors including C/EBPα and PPARγ. Insulin is an important factor for the induction and maintenance of adipocytes by directly activating PPARγ and C/EBPα expression through the PI3K-Akt1 signaling pathway [3,4]. Consequently, PPARγ and C/EBPα contribute to the expression of their targeting adipogenic genes, resulting in increased cell differentiation, fatty acid transportation, glucose uptake, and lipogenesis [3]. In particular, the activation of the lipogenesis pathway increases excessive synthesis and accumulation of triglyceride in lipid droplets of mature adipocytes, leading to hypertrophy or increasing fat-cell size [5]. Studies revealed that hypertrophic obesity and adipose cell size are causes of insulin resistance [2].

Experiments to provide insight into the molecular mechanisms controlling adipogenesis of human adipocytes revealed that intracellular calcium signaling inhibits the early phase of adipogenesis but stimulates the later phase and causes lipid filling [6]. Currently, attempts are being made to identify naturally occurring bioactive compounds that can suppress preadipocyte proliferation and adipogenesis [7].

Anthocyanins, a group of water-soluble polyphenolic compounds, are responsible for the red, purple, and blue color in fruits, vegetables, and plants. Anthocyanins have many health benefits such as antioxidant, anti-cancer, preventing cardiovascular disease, neuroprotective effect, anti-diabetes, and anti-obesity effects [8]. Studies show that anthocyanins possess anti-obesity activity through the regulation of adipocytes, including induction of cell cycle arrest, reduction of lipid accumulation by suppressing transcription factors expressions such as PPARγ, C/EBPα, C/EBPβ, C/EBPδ, and aP2, and promoting lipolysis [9]. Interestingly, anthocyanin-enriched extracts from fermented blueberry juice, cranberry, blue pea flower (*Clitoria ternatea*), and blueberry peel inhibits adipogenesis and lipogenesis in 3T3-L1 adipocytes via downregulation of adipogenic genes [10,11,12,13,14].

Riceberry rice (*Oryza sativa* L.), is a dark-purple rice originated from Hom Nin rice and Hom Mali 105 rice. The pigment from this rice contains anthocyanins which are cyanidin-3-*O*-glucoside (C3G) and peonidin-3-*O*-glucoside (P3G) [15,16]. An extract from Riceberry rice bran has a cytoprotective effect from oxidative damage [17], and prevents nephrotoxicity [18], as well as hepatotoxicity [19] in rats. In addition, it inhibits cancer cell proliferation and promotes apoptosis by inducing cell cycle arrest and DNA fragmentation, increasing p53 protein expression, decreasing caspase-3 protein expression [15,20]. Interestingly, Riceberry rice bran oil improves glycemic levels in diabetic rats by increasing the expression of glucose transporter 4 (Glut4) in muscle [16,21]. Studies with Riceberry rice extract (RBE) revealed that it inhibits key enzymes and steps of carbohydrate and lipid digestion and absorption [22]. Consumption of Riceberry rice bread reduces glycemic responses together with the improvement of antioxidant status in healthy subjects [23,24]. However, there is very limited information regarding the anti-obesity effect of anthocyanin-enriched extract from RBE.

The objective of this study is to investigate the effect of RBE on preadipocyte proliferation and adipogenesis in 3T3-L1 cells. We determined whether intracellular calcium signaling was involved in the RBE responses and examined its impact on transcription factors, adipogenic gene expression, and adipocyte function.

## 2. Materials and Methods

### 2.1. Materials

Mouse 3T3-L1 preadipocytes (CL-173™) were purchased from American Type Culture Collection (ATCC, Manassas, VA, USA). All reagents were purchased from Sigma Chemical Co. (St. Louis, MO, USA). The MUSE™ Cell Count and Viability and MUSE™ Cell Cycle kits were purchased from Merck (Millipore, Darmstadt, Germany). Triglyceride liquicolor GPO-POD kit was purchased from Human^®^ (Human, Wiesbaden, Germany). All gene-specific mouse primers were generated from IDT™ (Integrated DNA Technologies, Inc., Coralville, IA, USA). The TRIzol™, 2-(N-(7-Nitrobenz-2-oxa-1,3-diazol-4-yl)-amino)-2-Deoxyglucose (2-NBDG) and Dulbecco’s Modified Eagles Medium (DMEM)/high glucose were purchased from Invitrogen™ (Thermo Fisher, Waltham, MA, USA). The RQ1 DNase kit and reverse transcription system were purchased from Promega (Promega^®^, Madison, WI, USA). An iTaq™ Universal SYBR^®^ Green Supermix was purchased from Bio-Rad (Bio-Rad, Hercules, CA, USA).

### 2.2. Extraction of Riceberry Rice and Phytochemical Analysis

Riceberry rice harvested in Thailand was purchased from the local market. Briefly, the rice (2 kg) was extracted in 5 L of water at 50 °C for 40 min, then followed by freeze dryer lyophilization. Riceberry rice extract (RBE) was dissolved in distilled water (2 mg/mL) before use. Total phenolic content in RBE was determined using the Folin-Ciocalteau method with minor modification [25]. An aliquot of RBE (50 µL) was incubated with 50 µL of Folin-Ciocalteau reagent. After 5 min incubation in the dark, the mixture was incubated with 50 µL of 10% (*w*/*v*) sodium carbonate for 30 min. The absorbance of the mixture was measured at 760 nm. The total phenolic content was expressed as mg of gallic acid equivalents per g of extract (mg GAE/g extract). Total flavonoid content in RBE was determined as previously described [26]. Then, 100 µL of RBE was mixed with 30 µL of 5% (*w*/*v*) sodium nitrite and 400 µL of water. After 5 min incubation in the dark, 30 µL of 10% (*w*/*v*) aluminum chloride, 200 µL of 1 M sodium hydroxide, and 240 µL of water were added to each mixture. The absorbance was measured at 510 nm using a spectrophotometer. Total flavonoid content was expressed as mg of catechin equivalents per g of extract (mg CE/g extract). Quantification of total anthocyanin content in the extract was determined using a pH differential method [27]. RBE (500 µL) was incubated with 500 µL of two different buffer solutions including 0.025 M potassium chloride (pH 1.0) and 0.4 M sodium acetate (pH 4.5) for 15 min in the dark. The absorbance of each mixture was measured at 520 and 700 nm then calculated using the equation of A = (A520–A700) _pH1.0_–(A520–A700) _pH4.5_. The total anthocyanin content was expressed as mg of cyanidin-3-glucoside per g of extract (mg C3G/g extract).

### 2.3. HPLC

To quantify the major anthocyanins in RBE, high-performance liquid chromatography (HPLC) was performed using a C18 column (250 × 4.6 mm, 5 µm, Varian^®^) with minor modifications [28]. The extract (1 mg/mL) was dissolved in methanol with 2% (*v*/*v*) HCl solution. The absorbance was detected at 515 nm. Cyanidin-3-glucoside (C3G) and peonidin-3-glucoside (P3G) were used as standards. The results were expressed as µg of C3G or P3G per mg of extract.

### 2.4. UHPLC-ESI-Q-TOF-MS/MS

To characterize the phytochemical contents in RBE, liquid chromatography and tandem mass spectrometry (LC-MS/MS) were performed using a Titan C18 reverse phase column (50 × 21 mm, 1.9 µm particle size). The RBE was dissolved in a 0.1% formic acid solution to a final concentration of 10 mg/mL and filtered through a 0.22 µm Nylon syringe filter before injecting into the system at 0.3 mL/min flow rate for 20 min. The LC-MS/MS was carried out using an Ultimate 3000 UHPLC system (Thermo Scientific, Dionex, Sunnyvale, CA, USA) equipped with an Electrospray ionization-Quadrupole-Time of Flight Mass Spectrometer (ESI-Q-TOF-MS/MS; Model Impact II, Bruker Daltonik GmbH, Bremen, Germany). The HPLC gradients which included eluent A; 0.1% formic acid in water and eluent B; 0.1% formic acid in acetonitrile was run with the multistep linear gradient; 0–9 min: 5–30% B; 12–17 min: 95% B; 17.5 min: 5% B and held for 2 min. The column temperature was maintained at 30 °C and the injection volume was 5 and 10 µL for positive, and negative ionization mode, respectively.

The mass spectra were recorded under the following ESI inlet conditions: the capillary voltage of 3800 V for positive mode and 2500 V for negative mode, the scanning mass-to-charge (*m*/*z*) range of 50 to 1000, the pressure of the nebulizer at 2.0 bar, the drying gas temperature at 200 °C, and drying gas flow, 8.0 L/min. Automatic MS/MS experiments were performed adjusting the collision energy values 20–50 eV depending on *m*/*z* and using nitrogen as collision gas. Sodium formate solution was used as a calibrant for auto internal mass calibration.

The MS data were processed through Data Analysis 4.3 software (Bruker Daltonics, Bermen, Germany). The identification was performed by using MS-DIAL software (RIKEN, version 4.18) which matching experimental mass spectra against mass spectral libraries based on weighted similarity score of accurate mass and MS/MS spectra. Respect and GNPS mass spectral libraries were used and a cut off value of 80% was selected.

### 2.5. Cell Culture

**Figure 1:**
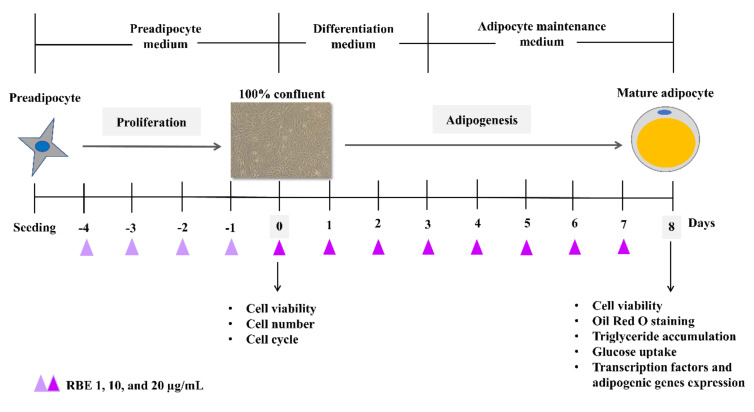
Schematic representation of 3T3-L1 differentiation into adipocytes.

For proliferation assay, mouse 3T3-L1 preadipocytes were cultured in Dulbecco’s Modified Eagles Medium (DMEM)/high glucose (HG) with 10% (*v*/*v*) heat-inactivated fetal bovine serum (FBS), 100 IU/mL penicillin, and 100 µg/mL streptomycin at 5% CO_2_ at 37 °C. Cells were seeded on 12-well plate (10,000 cells/mL) and cultured for 24 h followed by incubation with 1, 10, and 20 µg/mL RBE for 4 days. At this day, cells (control group) were grown in monolayer at 100% confluency. Cell viability, total cell number, and cell cycle were determined using MUSE™ Cell Count and Viability kit and MUSE™ Cell Cycle kit (Figure 1).

For cell differentiation, preadipocytes were seeded on 12 well plates (10,000 cells/mL) and cultured in DMEM/HG with 10% FBS, 100 IU/mL penicillin, and 100 µg/mL streptomycin at 5% CO_2_ at 37 °C. According to the proliferation experiment, cells were cultured for 4 days until 100% confluent. In early phase of adipogenesis, cells were induced by medium supplemented with 0.5 mM 3-isobutryl-1-methylxanthine (IBMX), 1 µM dexamethasone, and 2.5 µg/mL insulin for 3 days. In late phase of adipogenesis, cells were maintained in culture medium supplemented with 2.5 µg/mL insulin and incubated with 1, 10, and 20 µg/mL RBE until fully differentiation on day 8 (Figure 1). Cell viability, mature adipocyte number (Oil Red O staining), triglyceride levels, the glucose uptake activity, and mRNA expression of major transcription factors and adipogenic genes were then examined. All experiments were performed with 3T3-L1 cells from passages 4–16.

### 2.6. Cell Number and Viability

After 4 days of treatment, cells were trypsinized and resuspended in DMEM. A 20 µL of suspended cells were incubated with 380 µL of MUSE™ Cell Count and Viability reagent for 5 min in the dark. The mixture was loaded into the MUSE™ Cell Analyzer (Millipore) to quantify the number of viable and dead cells in each sample. The results were expressed as a percentage of cell viability (% of control) and total cell number (cells/mL).

After RBE treatment for 8 days, cells were trypsinized and resuspended in 1 mL DMEM and mixed with trypan blue in a 1:1 ratio and counted using a hemocytometer. The results were expressed as a percentage of cell viability (% of control) and total cell number (cells/mL).

### 2.7. Cell Cycle

After 4 days of treatment, the cell cycle of preadipocytes was determined using MUSE™ Cell Cycle kit (Millipore, Germany). Cells were trypsinized and resuspended in 1 mL PBS (pH 7.4) and fixed with 3 mL of 70% cooled ethanol at −20 °C overnight. After that, fixed cells were washed and resuspended two times in PBS. Then, the cell pellets were resuspended in 200 µL of MUSE™ Cell Cycle reagent and incubated for 30 min in the dark. Finally, the mixture was loaded into the MUSE™ Cell Analyzer (Millipore) to quantify the proportion of cells in each stage of the cell cycle. The results were expressed as the percentage of cells in G0/G1, S, and G2/M phase.

### 2.8. Real-Time Calcium Imaging Analysis

To obtain insight into the molecular mechanism of RBE, intracellular calcium recording was performed in 3T3-L1 cells [29]. Cells were cultured on round glass coverslips for 48 h until 90–100% confluent then incubated at 37 °C for 30 min with 2 µM Fura-2AM in calcium imaging buffer consisting of 136 mM NaCl, 4.8 mM KCl, 1.2 mM CaCl_2_, 1.2 mM MgSO_4_, 10 mM HEPES, 4 mM glucose, and 0.1% BSA at pH 7.4. The calcium signals were recorded using a dual excitation fluorometric imaging system (TILL-Photonics Grafelfingen, Gräfelfing, Germany) with excitation wavelength at 340 nm and 380 nm controlled by TILLvisION software. Fluorescence emissions were collected at 1 Hz, computed as an F340/F380 ratio, and expressed as the peak of calcium signals from individual cells. Ionomycin (1 µM) was used as a positive control.

### 2.9. Oil Red O Staining

Mature adipocytes were washed twice in PBS and fixed with 10% formalin overnight. Cells were washed twice with water and incubated with 60% isopropanol for 5 min. Then, cells were stained with Oil Red O solution for 20 min and the excess dye was washed five times with water. The stained lipid droplets were captured by an inverted microscope and the number of adipocytes quantified using ImageJ software. The results were expressed as total adipocyte count (cells/frame).

### 2.10. Determination of Triglyceride Level

Mature adipocytes were washed with PBS and lysed under sonication for 5 min following the previous study with modifications [30]. After centrifugation at 12,000× *g* for 10 min, 5 µL of supernatant was incubated with 250 µL of triglyceride reagent for 10 min in the dark. The absorbance was measured at 500 nm. The results were expressed as mg of triglyceride per mL (mg/mL).

### 2.11. Glucose Uptake

Glucose uptake was performed according to the previous method with slight modification [31]. After 8 days of incubation, mature adipocytes were incubated in PBS at 37 °C for 2 h, then incubated with 80 µM of fluorescent glucose analogue (2-NBDG) and 100 nM insulin at 37 °C for 60 min. The excess 2-NBDG was washed three times with ice-cold PBS. The fluorescence intensity of 2-NBDG was measured at 485 nm excitation wavelength and 535 nm emission wavelength using a fluorescence microplate reader. Data were normalized to the total protein concentration from the BCA kit (Thermo Fisher, USA) using BSA as a standard. The results were expressed as the percentage of glucose uptake (% of control).

### 2.12. Determination of mRNA Expression

At day 8 of differentiation, total RNA was extracted using TRIzol™ reagent (Invitrogen™, Thermo Fisher, USA). Quantification of RNA was determined using NanoDrop 1000 spectrophotometer. Total RNA (200 ng/µL) was treated with RQ1 DNase enzyme using RQ1 DNase treatment kit (Promega^®^, USA). After that, DNase-treated RNA was converted into cDNA using Reverse transcription system (Promega^®^, USA). Finally, 25 ng/µL of cDNA template was mixed with iTaq™ Universal SYBR^®^ Green Supermix (Bio-Rad, USA) and gene-specific mouse primers as shown in Table S1. RT-qPCR was carried out in a CFX384 TouchTM Real-Time PCR Detection system (Bio-RAD, CA, USA) using SYRB green detection according to the manufacturer’s instruction. The mRNA expression was normalized with β-actin using the 2^−ΔΔCt^ method. The result was expressed as the relative mRNA expression.

### 2.13. Determination of Akt1

To investigate the effects of RBE on Akt1 signaling in adipocytes, 3T3-L1 cells were cultured and differentiated in 6-well plate. After treatment with RBE for 8 days, cells were washed with cold PBS and lysed with 200 µL/well of ice-cold 1X MILLIPLEX^®^ MAP lysis buffer (EMD Millipore, Merck, Germany). Cell lysates were gently rocked for 15 min at 4 °C and centrifuged at 14,000× *g* under 4 °C for 15 min. Supernatants were collected and stored at −80 °C for further experiments. The phosphorylation levels of Akt1 (Ser473) were determined using the MILLIPLEX^®^ MAP Phospho/Total Akt1 2-plex Magnetic Bead Panel kit (EMD Millipore, Merck, Germany) according to the manufacturer’s instruction. The fluorescence intensity of the beads was measured and analyzed using the Luminex^®^ system (EMD Millipore, Merck, Germany). Data were normalized to the total protein concentration from the BCA kit (Thermo Fisher, USA) using BSA as a standard. The results were expressed as the Median Fluorescence Intensity (MFI) per mg protein (MFI/mg protein).

### 2.14. Lipolysis

Glycerol release represented the lipolysis rate and determined according to published method with minor modifications [32]. Mature adipocytes were starved in serum-free DMEM at 37 °C overnight. To obtain a basal control level, cells were treated with RBE only for 3 h. For lipolysis stimulation, cells were supplemented for 3 h with RBE and 100 nM isoproterenol. The media in each sample was incubated with free glycerol reagent (Sigma Aldrich, Germany) at 37 °C for 5 min. The absorbance was measured at 540 nm. The glycerol levels were calculated using a calibration curve of glycerol standard (0–260 µL/mL). Data were normalized to the total protein concentration from the BCA kit (Thermo Fisher, USA) using BSA as a standard. The results were expressed as mM of glycerol per mg protein (mM/mg protein).

### 2.15. Statistical Analysis

Data were expressed as mean ± standard error of the mean (SEM) from three independent experiments (*n* = 3). The statistical significance was analyzed using One-way analysis of variance (ANOVA) with Duncan’s post hoc test using SPSS version 22.0 software (SPSS Inc., Chicago, IL, USA). Statistical significance was established at *p* < 0.05.

## 3. Results

### 3.1. Phytochemical Composition in RBE

Total phenolic compounds, flavonoids, and anthocyanins in RBE were 63.33 ± 2.40 mg GAE/g extract, 18.00 ± 0.01 mg CE/g extract, and 10.13 ± 0.14 mg C3G/g extract, respectively. The detected anthocyanins were cyanidin-3-glucoside (2.05 ± 0.04 µg/mg extract) and peonidin-3-glucoside (0.78 ± 0.01 µg/mg extract). As reported in the previous study, RBE from acidic methanol with solid-phase extraction contained higher amount of total phenolic compounds, flavonoids, and anthocyanins than the obtained results from current study [22]. It may be because of different extracting solvents and methods. According to the chromatograms from UHPLC-MS/MS (Figure S1), seven compounds were identified based on their retention times, high-resolution mass spectra of the fragment ions, and compared to the previous study, including cyanidin-3-glucoside (C3G), peonidin-3-glucoside (P3G), petunidin-3-glucoside, caffeic acid, taxifolin, quercetin-3-rutinoside or rutin, and ferulic acid (Tables S2 and S3) [33].

### 3.2. RBE Inhibited Cell Proliferation of Preadipocytes

**Figure 2:**
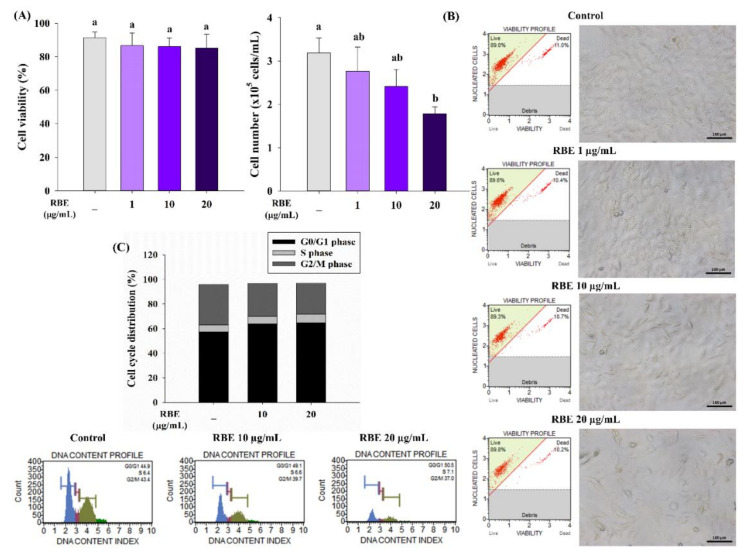
Effect of Riceberry rice extract (RBE) on 3T3-L1 preadipocyte proliferation during a 4-day period. (**A**) Treatment of cells with RBE did not affect cell viability. (**B**) RBE at 20 µg/mL significantly decreased the total cell number. Bright-field images (20× magnification) show the reduction in preadipocyte under RBE treatment compared to the control group. (**C**) RBE at 10 and 20 µg/mL induced cell cycle arrest with increased G0/G1 and decreased G2/M phases. The results are expressed as mean ± SEM from the three independent experiments. *p* < 0.05 for groups with different letters. Scale bars are 100 µm.

RBE (1–20 µg/mL) did not affect cell viability after 4 days of treatment (Figure 2A). The results also found that RBE at 20 µg/mL significantly reduced preadipocyte total cell number by 49% (Figure 2B). In addition, RBE at 10 and 20 µg/mL increased the proportion of cells at the G0/G1 phase with a concomitant decrease in the G2/M phase (Figure 2C).

### 3.3. Effect of RBE on Intracellular Calcium Signaling in Preadipocytes

**Figure 3:**
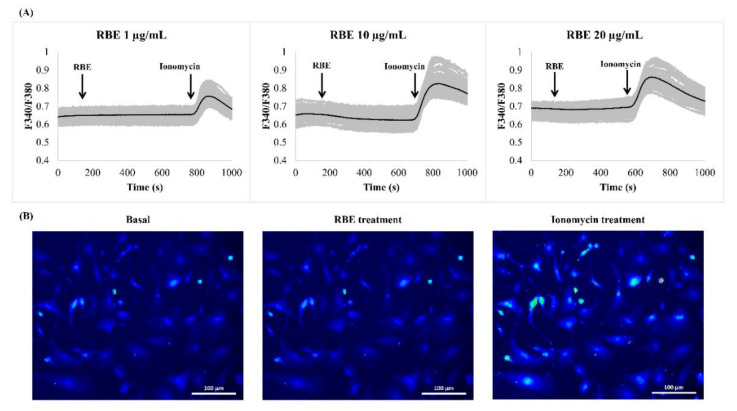
Effect of Riceberry rice extract (RBE) on intracellular calcium signals in 3T3-L1 cells. (**A**) Stimulation of cells with RBE 1, 10, and 20 µg/mL concentrations failed to induce calcium signals compared to 1 µM ionomycin as a positive control. Black lines represent average traces from all cells. Grey lines represent individual cell recordings. (**B**) Fluorescence emission from cells prior to, during RBE, and ionomycin treatments. Scale bars are 100 µm.

Since intracellular calcium signaling is known to control adipogenesis, we examined whether RBE utilized this signaling mechanism to exert its effect in preadipocytes. Stimulation of cells with RBE (1–20 µg/mL) failed to increase intracellular calcium signals compared to ionomycin treatment (positive control) (Figure 3A). The increases in cell fluorescence during RBE and ionomycin stimulation are shown in Figure 3B.

### 3.4. Effect of RBE on Adipogenesis

**Figure 4:**
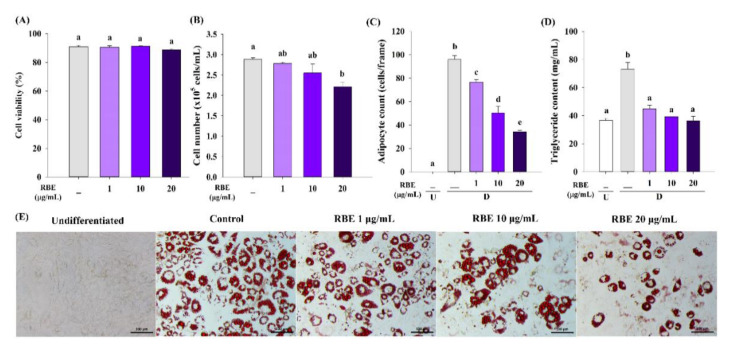
Effect of Riceberry rice extract (RBE) on adipogenesis in 3T3-L1 cells during an 8-day period. (**A**) Treatment of cells with RBE did not affect cell viability during adipogenesis. (**B**) RBE at 20 µg/mL significantly decreased the total adipocyte number. (**C**) RBE (1–20 µg/mL) decreased adipocyte number in a concentration-dependent manner. (**D**) A significant reduction in triglyceride accumulation was observed in mature adipocytes. (**E**) Treatment of cells with RBE inhibited adipogenesis by decreasing adipocyte number, cell size, and lipid droplet content as shown by Oil Red O staining (20× magnification). The results are expressed as mean ± SEM from the three independent experiments. Groups with a different letter represent statistical significance (*p* < 0.05). U: undifferentiated cells; D: differentiated cells. Scale bars are 100 µm.

In order to examine RBE’s effect on adipogenesis, preadipocytes were differentiated in adipogenic medium supplemented with RBE for 8 days. Treatment of cells with RBE significantly reduced the number of adipocytes in a concentration-dependent manner without affecting cell viability (Figure 4A,C). Moreover, RBE (20 µg/mL) significantly reduced the total adipocyte number by 23% (Figure 4B). In comparison with the control group, the level of triglyceride accumulation in adipocytes was significantly lowered by RBE (1–20 µg/mL) (Figure 4D). The morphological changes of stained adipocytes after exposure to adipogenic medium with RBE are shown in Figure 4E. Mature adipocytes demonstrated round-shape with lipid droplets in the cytoplasm compared to undifferentiated cells.

### 3.5. Effect of RBE on Transcription Factors and Akt1 Signaling in Late Phase of Adipogenesis

**Figure 5:**
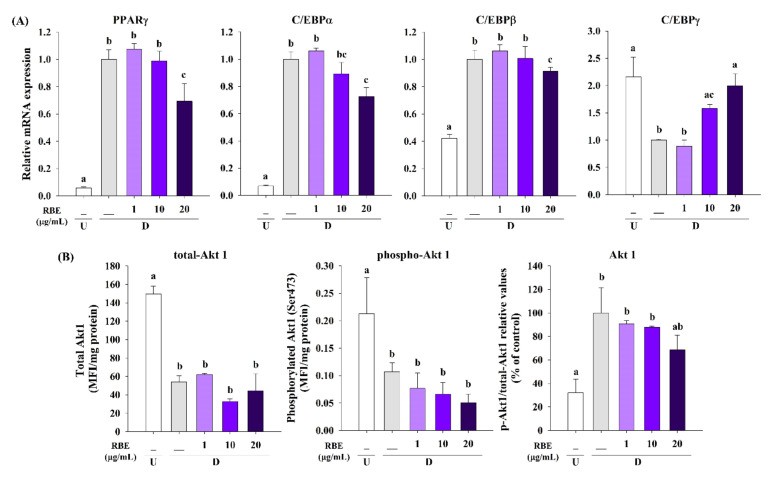
Effect of Riceberry rice extract (RBE) on adipogenic transcription factors and Akt1 signaling in 3T3-L1 cells. (**A**) RBE (20 µg/mL) significantly downregulated mRNA expression of PPARγ, C/EBPα, and C/EBPβ, and upregulated C/EBPγ in differentiated cells. (**B**) Treatment of cells with RBE tended to reduce the relative values of phospho-Akt1 (S473) to total-Akt1 in differentiated cells. The results are expressed as mean ± SEM from the three independent experiments. Groups with a different letter represent statistical significance (*p* < 0.05). Data were normalized with β-actin as an internal control. U: undifferentiated cells; D: differentiated cells.

After 8 days of adipogenic differentiation, the mRNA expression levels of PPARγ, C/EBPα, and C/EBPβ significantly increased in differentiated cells compared to undifferentiated control. Interestingly, RBE (20 µg/mL) significantly downregulated PPARγ, C/EBPα, and C/EBPβ mRNA expression but upregulated C/EBPγ mRNA expression (Figure 5A). In addition, differentiated cells demonstrated an increase in the relative value of p-Akt1 (Ser473)/total-Akt1, whereas RBE (20 µg/mL) tended to decrease its relative value (Figure 5B).

### 3.6. Effect of RBE on Adipogenic Gene Expression

**Figure 6:**
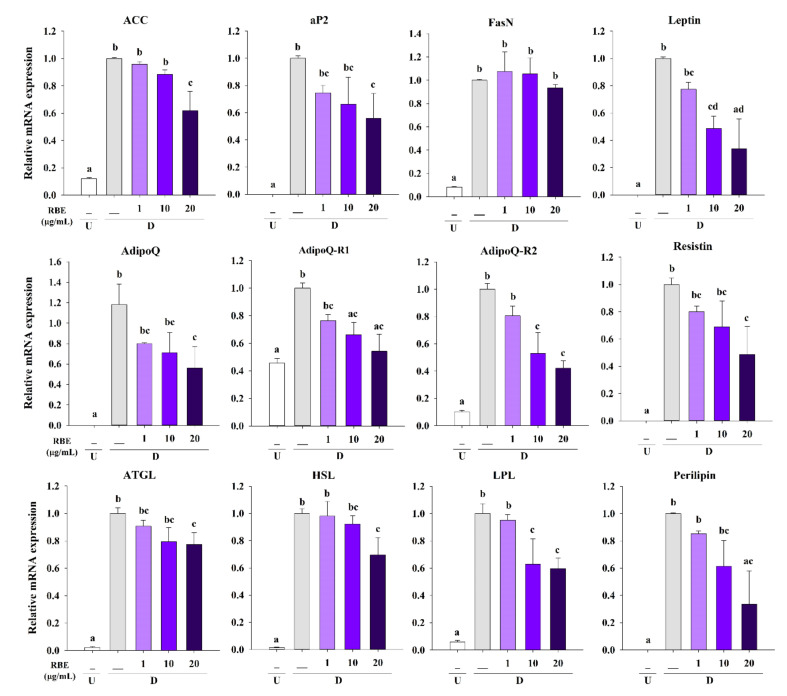
Effect of Riceberry rice extract (RBE) on adipogenic gene mRNA expression. Treatment with RBE (20 µg/mL) significantly downregulated adipogenic gene mRNA expression for ACC, aP2, Leptin, AdipoQ, AdipoQ-R1, AdipoQ-R2, Resistin, ATGL, HSL, LPL, and perilipin in differentiated cells. The results are expressed as mean ± SEM from the three independent experiments. Groups with a different letter represent statistical significance (*p* < 0.05). Data were normalized with β-actin as an internal control. U: undifferentiated cells; D: differentiated cells.

Based on the inhibitory effects of RBE on transcription factors and Akt1 signaling, its impact on adipogenic gene expression was determined. Treatment of cells with RBE (20 µg/mL) significantly downregulated the expression of adipogenic genes ACC, aP2, AdipoQ, leptin, resistin, perilipin, HSL, LPL, ATGL, and adiponectin receptors R1 and R2 (AdipoQ-R1 and AdipoQ-R2) compared to control differentiated cells in the absence of RBE (Figure 6).

### 3.7. Effect of RBE on Glucose Uptake, the Glut4 mRNA Expression, and Lipolysis

**Figure 7:**
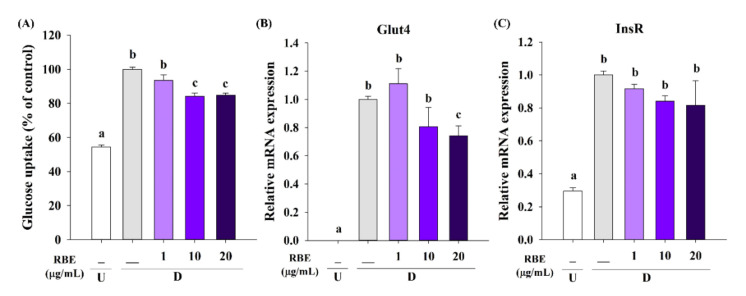
Effect of Riceberry rice extract (RBE) on glucose uptake in mature adipocytes. (**A**) RBE at 10 and 20 µg/mL significantly reduced glucose uptake into mature adipocytes after 8 days of treatment. (**B**) Downregulation of Glut4 mRNA expression in response to RBE at 20 µg/mL in differentiated cells. (**C**) Treatment of cells with RBE did not impact insulin receptor (InsR) mRNA expression. The results are expressed as mean ± SEM from the three independent experiments. Groups with a different letter represent statistical significance (*p* < 0.05). Data were normalized with β-actin as an internal control. U: undifferentiated cells; D: differentiated cells.

**Figure 8:**
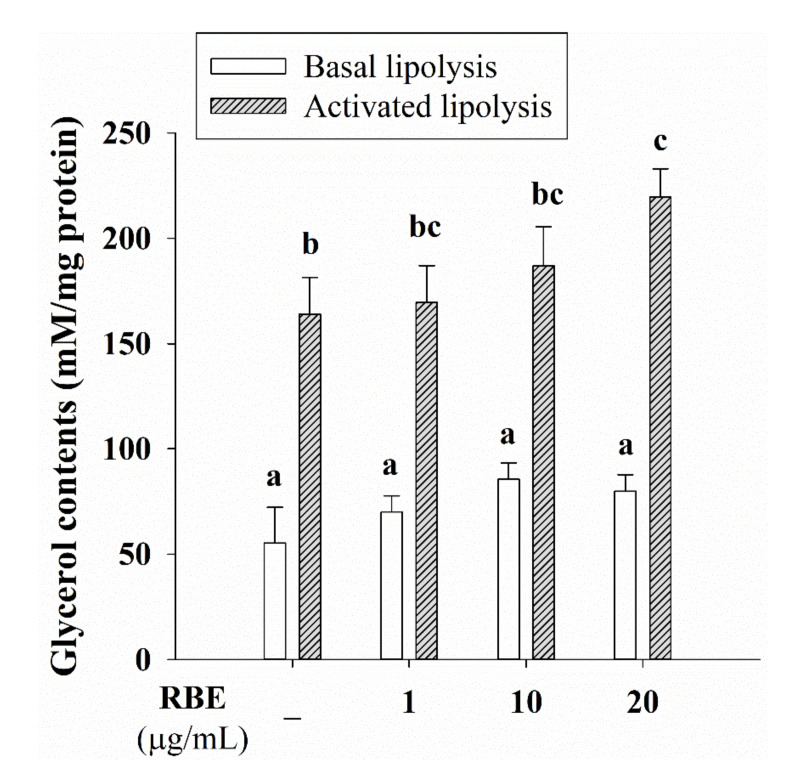
Effect of Riceberry rice extract (RBE) on lipolysis in adipocytes. RBE (20 µg/mL) significantly enhanced isoproterenol-induced glycerol release from adipocytes. The results are expressed as mean ± SEM from the three independent experiments. Groups with a different letter represent statistical significance (*p* < 0.05).

We examined whether RBE impacted glucose uptake in adipocytes and Glut4 gene expression. The results revealed that RBE at 10 and 20 µg/mL caused a 16% inhibition in glucose uptake compared to control untreated cells (Figure 7A). Moreover, RBE (20 µg/mL) treatment reduced Glut4 mRNA expression (Figure 7B) without affecting insulin receptor expression (Figure 7C). The experiments testing the effect of RBE on lipolysis revealed that it tended to increase the basal lipolysis, whereas significantly enhanced isoproterenol-induced glycerol release by 35%, compared to the control cells in the absence of RBE (20 µg/mL) (Figure 8).

## 4. Discussion

One of the most promising mechanisms by which natural products counteract obesity is by inhibiting adipogenesis, blocking preadipocyte proliferation and/or differentiation [2]. Aguilar et al. revealed that the inhibition of cell proliferation, by inducing the cell cycle arrest, can control adipogenesis [34]. In the present study, RBE demonstrated an anti-proliferative effect by reducing the number of viable preadipocytes without compromising cell viability. The results from flow cytometry assay indicated that treatment of 3T3-L1 preadipocytes with RBE inhibited cell proliferation by inducing cell cycle arrest at the G0/G1 and G2/M phases. During adipogenic differentiation, RBE also decreased the cell population which could be attributed to its sustained anti-proliferative effect. These findings are consistent with reports showing an anti-proliferative effect on 3T3-L1 preadipocytes by black soybean anthocyanins during adipocyte differentiation [35]. Real-time calcium imaging experiments, addressing the molecular mechanism, by which RBE inhibited cell proliferation and adipogenesis, revealed that calcium signals are not involved in its mechanism of action. These findings differ from studies demonstrating the importance of calcium signals for the adipocyte cell cycle and the early stages of adipogenesis [6,36,37]. Therefore, the mechanism of RBE might be the calcium-independent mechanisms as reported in previous studies, such as coffee extract inhibits preadipocyte proliferation and differentiation by interrupting the insulin signaling pathway by decreasing protein expression of the insulin receptor substrate 1 (IRS1) and further promoting their degradation [38].

Fibroblast-like preadipocytes begin to differentiate into round-shape mature adipocytes when exposed to adipogenic medium containing IBMX, dexamethasone, and insulin [5]. This process involves a cascade of transcription factors. Initially, there is the upregulation of transcription factor C/EBPβ by IBMX and dexamethasone during the early stage of adipocyte differentiation [39]. This is followed by PPARγ and C/EBPα gene expression, the master regulators of adipogenesis. Another member of the C/EBP family, C/EBPγ inhibits adipogenesis through heterodimerization and inactivation of C/EBPβ [39]. Our results revealed that RBE exerted the inhibitory effect on PPARγ and C/EBPα expression at least in part by upregulation C/EBPγ and downregulation of C/EBPβ during adipogenic differentiation. The activation of the PI3K-Akt signaling pathway by insulin enhances the expression of PPARγ and C/EBPα during preadipocyte differentiation [3]. Interestingly, RBE did not inhibit Akt1 phosphorylation, suggesting that the anti-adipogenic effects may not be attributed to the insulin-mediated PI3K-Akt signaling pathway. One alternative mechanism could be the downregulation of C/EBPβ with inhibition of the MEK-ERK and Akt1 pathways, leading to decreases in PPARγ and C/EBPα expression [38].

Apart from PPARγ and C/EBPα, adipocyte differentiation involves the expression of several genes critical for the development of the adipose phenotype and biosynthesis, including the lipogenic pathway, adipokine secretion, and insulin sensitivity [3]. In mature adipocytes, triglyceride synthesis is regulated by increasing lipogenic genes expression such as perilipin (lipid droplet-associated protein), fatty acid-binding protein (FABP or aP2), acetyl-CoA carboxylase (ACC, a fatty acid-synthesis enzyme), fatty acid synthase (FasN), lipoprotein lipase (LPL), hormone-sensitive lipase (HSL), and adipose triglyceride lipase (ATGL) [5,40]. In addition, it requires the expression of important adipokines genes (adiponectin, leptin, and resistin) [41] and glucose transporter 4 (Glut4) for glucose uptake into mature adipocytes [3]. Consistent with the Oil Red O staining and triglyceride accumulation experiments, RBE significantly decreased the expression of lipogenic genes including perilipin, aP2, ACC, LPL, HSL, and ATGL. Adipokine marker expression profiles (AdipoQ, AdipoQ-R1, AdipoQ-R2, leptin, and resistin) were also reduced by RBE treatment during adipogenesis. Furthermore, RBE suppressed Glut4 gene expression, leading to decrease glucose uptake in adipocytes. The suppression of adipogenic gene expression by RBE inhibited adipocyte proliferation and differentiation as well as downregulation of key transcription factors. These findings are consistent with studies where anthocyanin-rich plants, such as cranberry and blue pea flower extract, are capable of inhibiting adipogenesis by downregulating adipogenic transcription factors and their targets [11,12,14].

Lipolysis catalyzes the breakdown of triglyceride in intracellular lipid droplets, leading to reduce the fat-cell size and hypertrophic adipocytes [42]. Generally, there are two processes of lipolysis: basal lipolysis and activated lipolysis by catecholamine [42]. Isoproterenol or catecholamines bind to the β3-adrenergic receptor on adipocytes and promote lipolysis through the cAMP-PKA signaling pathway [42]. Our study revealed that RBE had a tendency to enhance catecholamine-stimulated lipolysis in 3T3-L1 cells that is consistent with anthocyanin-rich *Clitoria ternatea* flower petal extract [14]. This finding suggests a beneficial effect of RBE on catecholamine-induced lipolysis in mature adipocytes.

Several reports show that phytochemical compounds in plants can inhibit adipogenesis by downregulating adipogenic transcription factors in preadipocytes. In particular, black rice (*Oryza sativa* L.) extract inhibited adipogenesis by downregulating the mRNA expression of PPARγ, C/EBPα, LPL, and aP2 in mesenchymal C3H10T1/2 cells [43]. Jang et al. reported that C3G and P3G present in black rice extract are promising anti-adipogenic compounds [43]. Apart from anthocyanins, phenolic compounds in RBE, including quercetin-3-rutinoside or rutin, caffeic acid, and ferulic acid could have similar action as adipogenic inhibitors [44]. Among these phenolic compounds, rutin expressed the highest inhibitory activity on lipid accumulation in 3T3-L1 cells followed by caffeic acid, and ferulic acid, respectively [44]. Rutin suppressed the adipocyte differentiation by downregulating the expression of PPARγ, C/EBPα, FasN, LPL, and aP2 [45,46]. Moreover, coffee extract which mainly contains caffeic acid showed the anti-adipogenic effects by suppressing the expression of adipocyte marker genes including PPARγ, C/EBPα, aP2, LPL, Glut4 and adiponectin [47]. Interestingly, ferulic acid binds to PPARγ leading to conformational changes and decreases PPARγ-target genes expression including aP2, FasN, LPL, perilipin1, and adiponectin [48]. The binding to PPARγ by phenolic compounds may be responsible for inhibition of adipocyte differentiation. Our findings suggest that anthocyanins (C3G and P3G) and phenolic compounds (rutin, caffeic acid, and ferulic acid) in RBE could be responsible for its anti-adipogenic effect.

## 5. Conclusions

**Figure 9:**
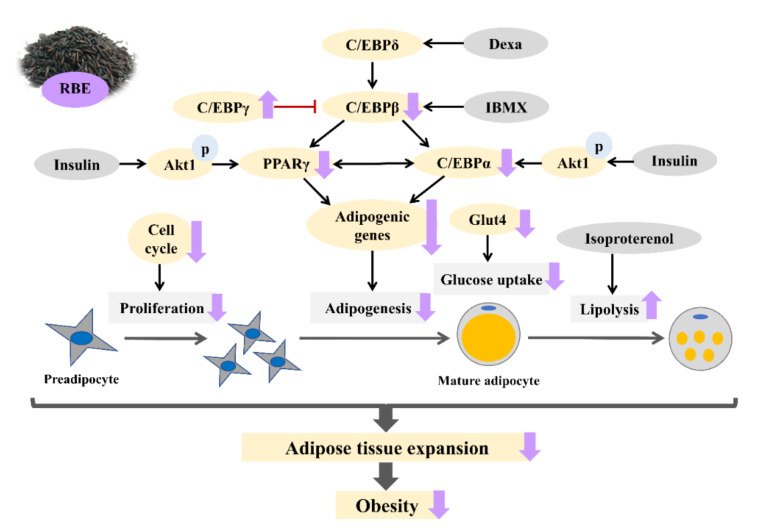
Schematic summary of the inhibitory effect of Riceberry rice extract (RBE) on cell proliferation and differentiation in 3T3-L1 adipocytes. RBE suppressed preadipocyte proliferation through inducing cell cycle arrest. RBE downregulated adipogenic transcription factors and their target genes, leading to inhibit adipogenesis and reduce glucose uptake. In addition, RBE enhanced isoproterenol-induced lipolysis in mature adipocytes.

This study suggested that anthocyanin-enriched RBE inhibited cell proliferation and differentiation in 3T3-L1 cells (Figure 9). In addition, RBE blocked the early stage of adipocyte proliferation by inducing cell cycle arrest at G0/G1 and G2/M phases. In the process of cell differentiation, RBE upregulated transcription factors C/EBPγ with concomitant downregulation of major transcription factors including C/EBPβ, PPARγ, and C/EBPα and their target genes such as ACC, aP2, LPL, HSL, ATGL, adiponectin, leptin, resistin, and perilipin. This resulted in decreased triglyceride accumulation in adipocytes. Moreover, RBE reduced glucose uptake by downregulating Glut4 mRNA expression and enhanced catecholamine-induced lipolysis in mature adipocytes. These findings suggest a potential application for RBE in the treatment of obesity and related diseases.

## Supplementary Materials

The following are available online at https://www.mdpi.com/2072-6643/12/8/2480/s1, Figure S1: Extracted ion chromatogram of phytochemical compounds detected in Riceberry rice extract (RBE) obtained by UHPLC–ESI-Q-TOF-MS/MS in negative and positive ion mode, Table S1: List of primers for RT-qPCR, Table S2: MS and MS/MS data of phytochemical compounds detected in Riceberry rice extract (RBE) obtained by UHPLC–ESI-Q-TOF-MS/MS in negative mode, Table S3: MS and MS/MS data of phytochemical compounds detected in Riceberry rice extract (RBE) obtained by UHPLC–ESI-Q-TOF-MS/MS in positive mode.

## Abbreviations

ACC	acetyl CoA carboxylase
AdipoQ	adiponectin
AdipoQ-R1,R2	adiponectin receptor-1,2
aP2/Fabp4	fatty acid binding protein 4
ATGL	adipose triglyceride lipase
β-actin	Beta-actin
C3G	cyanidin-3-*O*-glucoside
[Ca^2+^]_i_	intracellular calcium concentration
CE	catechin equivalent
C/EBPα,β,γ	CCAAT-enhancer binding protein-alpha, beta, gamma
FasN	fatty acid synthase
Fura-2AM	Fura-2 acetoxymethyl ester
GAE	gallic acid equivalent
Glut4	glucose transporter 4
HSL	hormone-sensitive lipase
IBMX	3-isobutyl-1-methylxanthine
InsR	insulin receptor
LPL	lipoprotein lipase
2-NBDG	2-(*N*-(7-Nitrobenz-2-oxa-1,3-diazol-4-yl) amino)-2-Deoxyglucose
P3G	peonidin-3-*O*-glucoside
PPARγ	peroxisome proliferator-activated receptor-gamma
RBE	Riceberry rice extract
UHPLC-ESI-Q-TOF-MS/MS	ultra-high performance liquid chromatography coupled with electrospray ionization-quadrupole-time of flight-mass spectrometry
