# Supplementary material for: Anthocyanin-Enriched Riceberry Rice Extract Inhibits Cell Proliferation and Adipogenesis in 3T3-L1 Preadipocytes by Downregulating Adipogenic Transcription Factors and Their Targeting Genes
Source: Nutrients. 2020 Aug 17;12(8):2480. doi: 10.3390/nu12082480 (PMC7469062; doi:10.3390/nu12082480)
Supplement: Supplementary file 1 [file nutrients-12-02480-s001.pdf]

ONLINE SUPPORTING MATERIAL

Anthocyanin-Enriched Riceberry Rice Extract Inhibits Cell Proliferation and Adipogenesis in 3T3-L1 Preadipocytes by Downregulating Adipogenic Transcription Factors and Their Targeting Genes

Phutthida Kongthitilerd, Tanyawan Suantawee, Henrique Cheng, Thavaree Thilavech, Marisa Marnpae and Sirichai Adisakwattana

Figure S1……………………………………………………………………….. 2

Table S1…………………………………………………………………………3

Table S2…………………………………………………………………………4

Table S3…………………………………………………………………………4

Figure S1. Extracted ion chromatogram of phytochemical compounds detected in riceberry rice extract (RBE) obtained by UHPLC–ESI-Q-TOF-MS/MS in negative (a) and positive (b) ion mode.

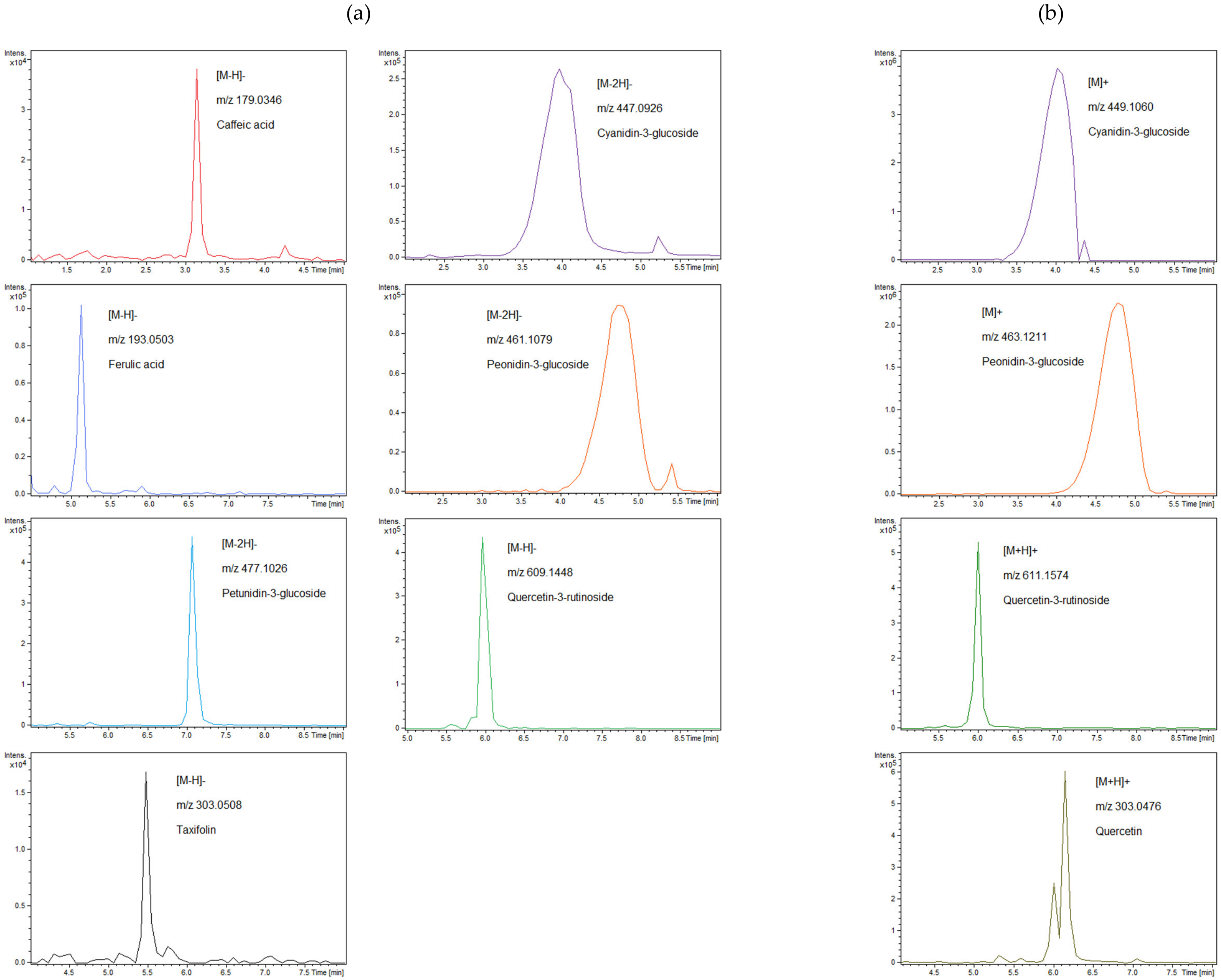

Table S1. List of primers for RT-qPCR

Gene	Forward primer (5′-3′)	Reverse primer (5′-3′)	Size (bp)
**PPARγ**	ACGTGCAGCTACTGCATGTGA	AGAAGGAACACGTTGTCAGCG	125
**C/EBPα**	GGAACTTGAAGCACAATCGATC	TGGTTTAGCATAGACGTGCACA	156
**C/EBPβ**	TGAGCGACGAGTACAAGATGC	GACAGCTGCTCCACCTTCTTCT	154
**C/EBPγ**	ATCGACTTCAGCGCCTACAT	GCTTTGTGGTTGCTGTTGAA	101
**InsR**	CAGAAGCACAATCAGAGTGAGTATGAC	ACCACGTTGTGCAGGTAATCC	137
**AdipoQ**	GCACTGGCAAGTTCTACTGCAA	GTAGGTGAAGAGAACGGCCTTGT	122
**AdipoQ-R1**	CCCCCTTACCCCCGTCCTTAC	GGCGTGGCTTTGTTTGTCCTA	121
**AdipoQ-R2**	TGCGCACACGTTTCAGTCTCCT	TTCTATGATCCCCAAAAGTGTGC	156
**Leptin**	GAGACCCCTGTGTCGGTTC	CTGCGTGTGTGAAATGTCATTG	139
**Resistin**	TGCCAGTGTGCAAGGATAGACT	CGCTCACTTCCCCGACAT	80
**aP2**	AAGGTGAAGAGCATCATAACCCT	TCACGCCTTTCATAACACATTCC	133
**FasN**	AGGTGGTGATAGCCGGTATGT	TGGGTAATCCATAGAGCCCAG	138
**ACC**	CGGACCTTTGAAGATTTTGT	GCTTTATTCTGCTGGGTGAA	233
**Glut4**	CAACTGGACCTGTAACTTCATTGT	ACGGCAAATAGAAGGAAGACGTA	87
**HSL**	GCTGGAGGAGTGTTTTTTTGC	AGTTGAACCAAGCAGGTCACA	64
**ATGL**	GGATGAAAGAGCAGACGGGTAG	CGCAAGACAGTGGCACAGAG	144
**LPL**	GGCTCTGCCTGAGTTGTAGAA	GGCATCTGAGAGCGAGTCTTC	112
**Perilipin**	AAGGATCCTGCACCTCACAC	CCTCTGCTGAAGGGTTATCG	101
**β-actin**	TGTCCACCTTCCAGCAGATGT	AGCTCAGTAACAGTCCGCCTAGA	101

PPARγ: peroxisome proliferator-activated receptor-gamma; C/EBPα,β,γ: CCAAT-enhancer binding protein-alpha, beta, gamma; InsR: insulin receptor; AdipoQ: adiponectin; AdipoQ-R1,R2: adiponectin receptor-1,2; aP2/Fabp4: fatty acid binding protein 4; FasN: fatty acid synthase; ACC: acetyl CoA carboxylase; Glut4: glucose transporter 4; HSL: hormone-sensitive lipase; ATGL: adipose triglyceride lipase; LPL: lipoprotein lipase; β-actin: Beta-actin.

Table S2. MS and MS/MS data of phytochemical compounds detected in riceberry rice extract (RBE) obtained by UHPLC–ESI-Q-TOF-MS/MS in negative mode

No	RT (min)	Compounds	Molecular Formula	Adduct type	Patent ion (m/z)	MS/MS fragments (m/z)
1	3.14917	Caffeic acid	$C_9H_8O_4$	[M-H]-	179.0346	135.0448, 134.0369, 136.0483, 179.0337, 107.0504, 117.0351, 137.0489, 151.0035, 133.0268, 107.0143
2	3.97533	Cyanidin-3-glucoside	$C_{21}H_{21}O_{11}$	[M-2H]-	447.0926	284.032, 285.0389, 286.0423, 447.0923, 299.0554, 125.024, 448.0953, 287.0462, 283.0253, 147.0081
3	4.73268	Peonidin-3-glucoside	$C_{22}H_{23}O_{11}$	[M-2H]-	461.1079	299.0551, 298.0477, 300.0587, 283.0241, 284.0319, 285.0382, 327.1456, 301.0648, 257.0455, 459.1878
4	5.14577	Ferulic acid	$C_{10}H_{10}O_4$	[M-H]-	193.0503	134.0372, 178.0269, 133.0295, 135.0406, 179.0302, 149.0598, 193.0505, 137.0236, 106.0424, 136.0426 125.0241
5	5.49005	Taxifolin	$C_{15}H_{12}O_7$	[M-H]-	303.0508	217.0495, 175.0407, 285.0401, 178.9985, 151.0038, 153.0186, 151.0403, 177.0199, 181.0141
6	5.97192	Quercetin-3-rutinoside	$C_{27}H_{30}O_{16}$	[M-H]-	609.1448	609.145, 300.0271, 301.0342, 610.1486, 611.1507, 302.0377, 178.9983, 343.045, 151.0031, 612.153
7	7.07342	Petunidin-3-glucoside	$C_{22}H_{23}O_{12}$	[M-2H]-	477.1026	477.1026, 314.0424, 478.1059, 315.0475, 479.1081, 357.0608, 286.0472, 271.0239, 285.0405, 316.0499

Table S3. MS and MS/MS data of phytochemical compounds detected in riceberry rice extract (RBE) obtained by UHPLC–ESI-Q-TOF-MS/MS in positive mode

No	RT (min)	Compounds	Molecular Formula	Adduct type	Patent ion (m/z)	MS/MS fragments (m/z)
1	4.02072	Cyanidin-3-glucoside	$C_{21}H_{21}O_{11}$	[M]+	449.1060	287.0533, 288.0568, 289.059, 290.0618
2	4.77668	Peonidin-3-glucoside	$C_{22}H_{23}O_{11}$	[M]+	463.1211	301.0687, 302.0722, 286.0453, 303.0744, 287.0491, 258.0507, 304.0767, 259.0563
3	6.01357	Quercetin-3-rutinoside	$C_{27}H_{30}O_{16}$	[M+H]+	611.1574	303.0473, 304.0509, 85.0275, 129.053, 305.0525, 145.048, 127.0374, 97.027, 147.0636, 151.0375
4	6.15097	Quercetin	C15H10O7	[M+H]+	303.0476	303.0475, 229.0477, 304.051, 153.0166, 257.0422, 137.0218, 165.0166, 285.0374, 201.053, 247.0578
